# Hyperoxia Elevates Adrenic Acid Peroxidation in Marine Fish and Is Associated with Reproductive Pheromone Mediators

**DOI:** 10.3390/md13042215

**Published:** 2015-04-14

**Authors:** Ming Long Sirius Chung, Jean-Marie Galano, Camille Oger, Thierry Durand, Jetty Chung-Yung Lee

**Affiliations:** 1 School of Biological Sciences, the University of Hong Kong, Pokfulam Road, Hong Kong; E-Mails: sirius09@hku.hk (M.L.S.C.); jettylee@hku.hk (J.C.-Y.L.); 2 Institute of Biomolecules Max Mousseron (IBMM), UMR 5247 CNRS, ENSCM, University of Montpellier, F-34093 Montpellier cedex 05, France; E-Mails: jgalano@univ-montp1.fr (J.-M.G.); camille.oger@univ-montp1.fr (C.O.); thierry.durand@univ-montp1.fr (T.D.)

**Keywords:** adrenic acid, dihomo-isofurans, dihomo-isoprostanes, medaka

## Abstract

The development of oxidative stress in the marine ecosystem is a concurring concern in fish reproductive behavior. Marine fish being rich in polyunsaturated fatty acids (PUFA) are precursors of prostaglandin pheromone mediators but also vulnerable to lipid peroxidation. It is yet to be determined if hypoxia or hyperoxia environment, a cumulative effect in the marine ecosystem affect pheromone mediators in fish, and to understand if this is associated with the generation of oxidized lipid products of PUFA. Novel oxidized lipid metabolites, isoprostanoids (15-F_2t_-isoprostane, 7(*RS*)-7-F_2t_-dihomo-isoprostane, 17(*RS*)-17-F_2t_-dihomo-isoprostane, 8-F_3t_-isoprostane, 4(*RS*)-4-F_4t_-neuroprostane, 10-F_4t_-neuroprostane), isofuranoids (isofurans, 10-*epi*-17(*RS*)-SC-Δ^15^-11-dihomo-isofuran and neurofurans), hydroxyeicosatetraenoic acids and resolvins, PUFA (arachidonic, adrenic, eicosapentaenoic and docosahexaenoic acids) and prostaglandin pheromone mediators in fish muscle were determined in marine male and female fish muscles before and after interaction in a hypoxia or hyperoxia environment. Reproductive behaviors were also assessed. Our study showed oxidized lipid metabolites of arachidonic, eicosapentaenoic, and docosahexaenoic acids were not influenced by hypoxia and hyperoxia exposure in the fishes and no gender differences were found. However, adrenic acid and its oxidized products, 17(*RS*)-17-F_2t_-dihomo-isoprostane and 10-*epi*-17(*RS*)-SC-Δ^15^-11-dihomo-isofuran showed strong correspondence with male fish pheromone mediators and reproductive behavior when under oxidative stress especially, hyperoxia. The occurrence of hypoxia and hyperoxia in the marine ecosystem may not be detrimental to marine fish and instead presents as being beneficial in reproductive behavior.

## 1. Introduction

The occurrence of oxidative stress by means of hypoxia and hyperoxia is a common phenomenon in the marine ecosystem and under adverse conditions such as climate change and environmental contaminants. Hypoxia condition in the marine ecosystem leads to excessive growth of some toxic algae and disrupts sex hormone concentrations [1]. An overgrowth of these algae leads to death and accumulation of organic matter by bacterial decomposition, and consequently causes oxygen depletion [1,2,3]. Hyperoxia environment is a natural occurrence of photosynthetic organisms and the presence of algae in the aquatic environment upon sunlight exposure elevates the rates of oxygen production and excessive reactive oxygen species (ROS) accumulation [4,5].

Despite marine fish being rich in polyunsaturated fatty acids (PUFA), in particular docosahexaenoic acid (DHA) and eicosapentaenoic acid (EPA), it is unclear if hypoxia and hyperoxia conditions lead to lipid peroxidation. Exposure to chemical oxidants of marine fish acutely depleted PUFA and prostaglandin-F_2α_ (PGF_2α_) from arachidonic acid (AA), and elevated non-enzymatic oxidized lipid products mainly related to PGF_2α_ namely F_2_-isoprostanes from AA, F_3_-isoprostanes from EPA, F_4_-isoprostanes (also related as F_4_-neuroprostanes) from DHA, and lipoxygenase-dependent lipid products [6]. However, PGF_2α_ and its derivatives, 15-keto-PGF_2α_ and 13,14-dihydro-15-keto-PGF_2α_ are also mediators for pheromone production in fish muscles [7,8,9,10]. These pheromones are chemicals secreted externally by one individual and received by a second individual. They serve for communication within species and may affect the behavior and developmental process. The release of these pheromones can stimulate the central nervous system and hence elevate the reproductive behavior of the fish [11]. Insufficient study has been made to evaluate the effect and the role of these PUFAs and the oxygenated metabolites in marine fish development. It is not only the yield of important essential PUFA in fish that may be affected by environmental oxidative stress conditions, but it could impose physiological change in fish behavior and alter reproduction [1]. We investigated if hypoxia and hyperoxia conditions modify lipid peroxidation of omega-3 PUFA (DHA and EPA) and omega-6 PUFAs (AA and adrenic acid, AdA), as well as the effect of eicosanoid pheromone production in marine fish muscle. 

## 2. Results 

### 2.1. Effect of Oxidative Stress on Eicosanoid Pheromone Mediators

**Figure 1:**
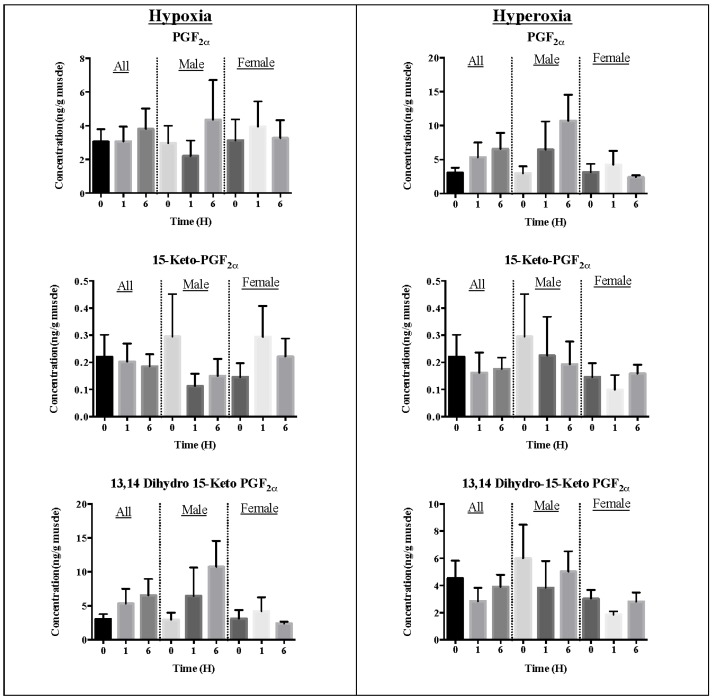
Concentrations of prostaglandin pheromone mediators determined in fish muscles under oxidative stress. Levels are mean ± SEM, *n* = 8 for all and *n* = 4 for each gender group.

Exposure to hypoxia or hyperoxia to the fishes did not affect the generation of the pheromone mediator PGF_2α_ and the derivatives 15-keto-PGF_2α_ and 13,14-dihydro-15-keto-PGF_2α_ (Figure 1) in fish muscles. The levels of pheromones did not show any differences between male and female fishes under all treatment conditions. 

### 2.2. Effect of Oxidative Stress on Fatty Acids

**Figure 2:**
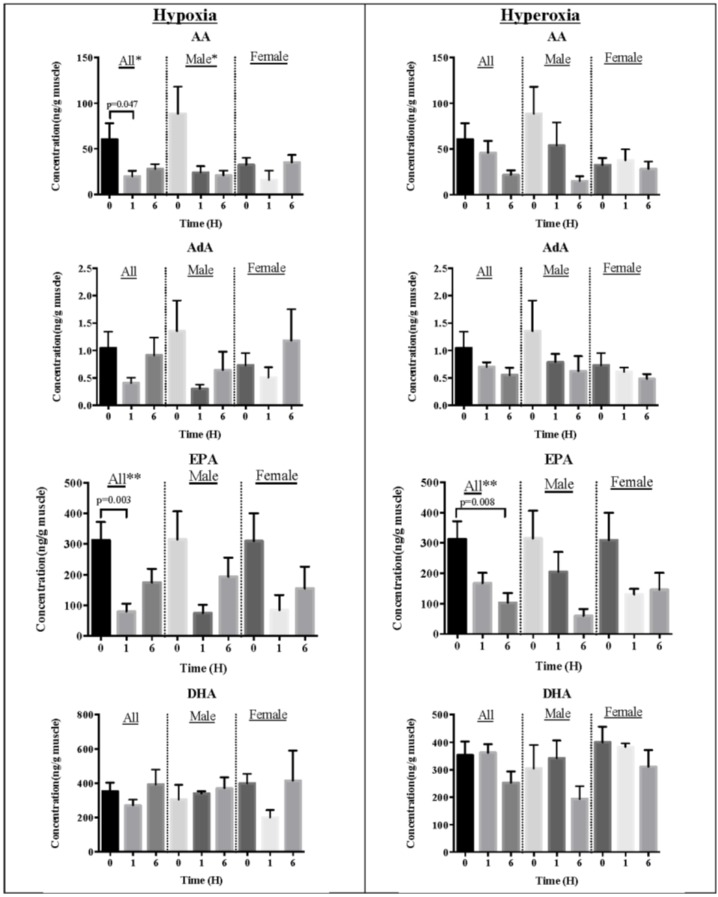
Concentrations of polyunsaturated fatty acids determined in fish muscles under oxidative stress. Annotations indicate AA: arachidonic acid; AdA: adrenic acid; EPA: eicosapentaenoic acid; DHA: docosahexaenoic acid. Levels are mean ± SEM, *n* = 8 for all and *n* = 4 for each gender group. Only significant *p*-values tested by unpaired Student’s *t*-test are annotated in the graphs. ANOVA indicates * *p* < 0.05 and ** *p* < 0.01 between treatment and time.

The impact of oxidative stress on omega-3 PUFAs (DHA and EPA) and omega-6 PUFAs (AA and AdA) was not distinct. Hypoxia exposure for 1 h lowered AA and EPA levels but not DHA and AdA in fish muscle compared to normoxia, and no effect was found after six hours exposure for all the studied PUFAs (Figure 2). The exposure of one hour hyperoxia had no effect on all PUFAs compared to normoxia but after six hours, only EPA reduced (Figure 2) in our study. We found only in hypoxia environment showed decreased AA in male fish but no difference was found for other PUFAs between male and female fish under all treatment conditions.

### 2.3. Effect of Oxidative Stress on Generation of Lipid Peroxidation Products 

#### 2.3.1. Enzyme-Independent

Both hypoxia and hyperoxia treatments did not significantly change levels of oxidized products of AA namely 15-F_2t_-isoprostane, 2,3-dinor-15-F_2t_-isoprostane and 2,3-dinor-5,6-dihydro-15-F_2t_-isoprostane in fish muscles after one hour and six hours exposure compared to normoxia (Table 1). Hypoxia treatment also did not show any effect on the release of the oxidized products, particularly 7(*RS*)-7-F_2t_-dihomo-isoprostane and 17(*RS*)-17-F_2t_-dihomo-isoprostane from AdA, 8-F_3t_-isoprostane from EPA, and 4(*RS*)-4-F_4t_-neuroprostane and 10-F_4t_-neuroprostane from DHA compared to normoxia in fish muscles after one hour and six hours exposure. In hyperoxia environment, the release of these oxidized products did not alter compared to normoxia after one hour and six hours exposure. Furthermore in normoxia, female fish muscle had higher 10-F_4t_-neuroprostane than male and no difference was found between the genders under other treatment conditions (Table 1).

#### 2.3.2. Enzyme-Dependent

Oxidized lipid products from AA could be released via lipoxygenase enzyme (LOX) to give 5(*S*)-, 8(*S*)-, 12(*S*)- and 15(*S*)-hydroxyeicosatetraenoic acid (HETE), from EPA to give resolvin E1 (RvE1) and DHA to give resolvin D1 (RvD1). Only hypoxia exposure showed significant increased 5(*S*)-HETE levels in male fishes and no gender difference was found between male and female fishes on other HETE levels under all treatment conditions. Aside from 15(*S*)-HETE being elevated significantly after six hours hyperoxia exposure, both hypoxia or hyperoxia treatment did not alter all the HETEs compared to normoxia in fish muscle (Table 2). Hyperoxia condition altered RvE1 levels in the fish muscle and no effect was found for RvD1 levels. There was no gender difference in RvD1 and RvE1 levels in both treatments. 

**Figure 3:**
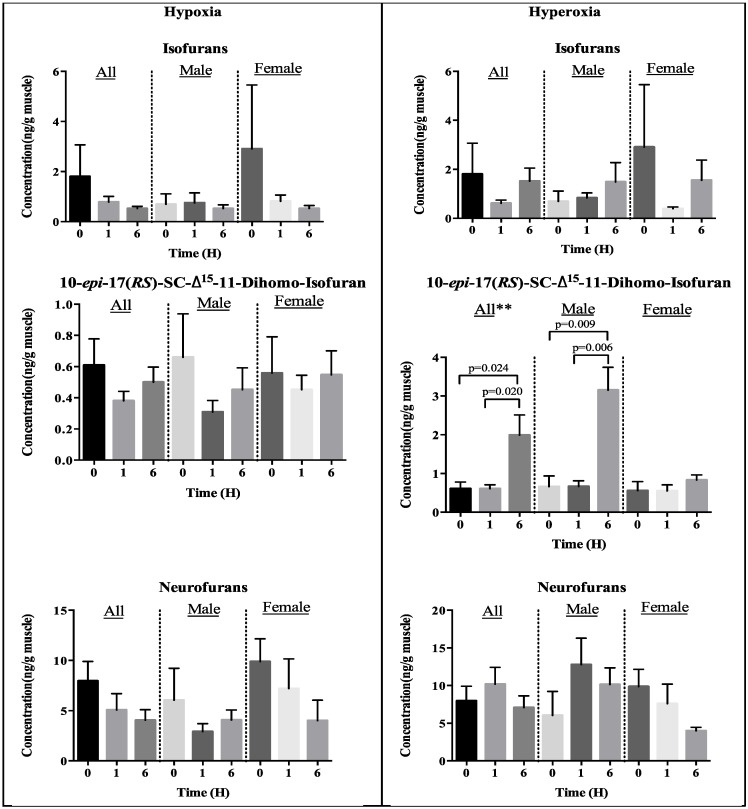
Concentrations of isofuranoids determined in marine fish muscles under oxidative stress. Sources of isofurans are from AA, 10-*epi*-17(*RS*)-SC-Δ^15^-11-dihomo-isofuran from AdA and neurofurans from DHA. Levels are mean ± SEM, *n* = 8 for all and *n* = 4 for each gender group. Only significant *p*-values tested by unpaired Student’s t-test are annotated in the graphs. ANOVA indicates ** *p* < 0.01 between treatment and time.

As displayed in Figure 3 both hypoxia and hyperoxia treatments did not affect the generation of isofurans and neurofurans in fish muscles compared to normoxia. Moreover, these levels showed no difference between male and female fishes at normoxia, and even when exposed to hypoxia and hyperoxia conditions. However, it was intriguing to find hyperoxia exposure substantially augmented 10-*epi*-17(*RS*)-SC-Δ^15^-11-dihomo-isofuran level after six hours exposure compared to normoxia environment, but not for hypoxia. It was identified that the elevation was significantly contributed by male fishes and not by female fishes (Figure 3).

### 2.4. Correlation between PUFA and Oxidized Lipid Products, and Pheromone Mediators

**Figure 4:**
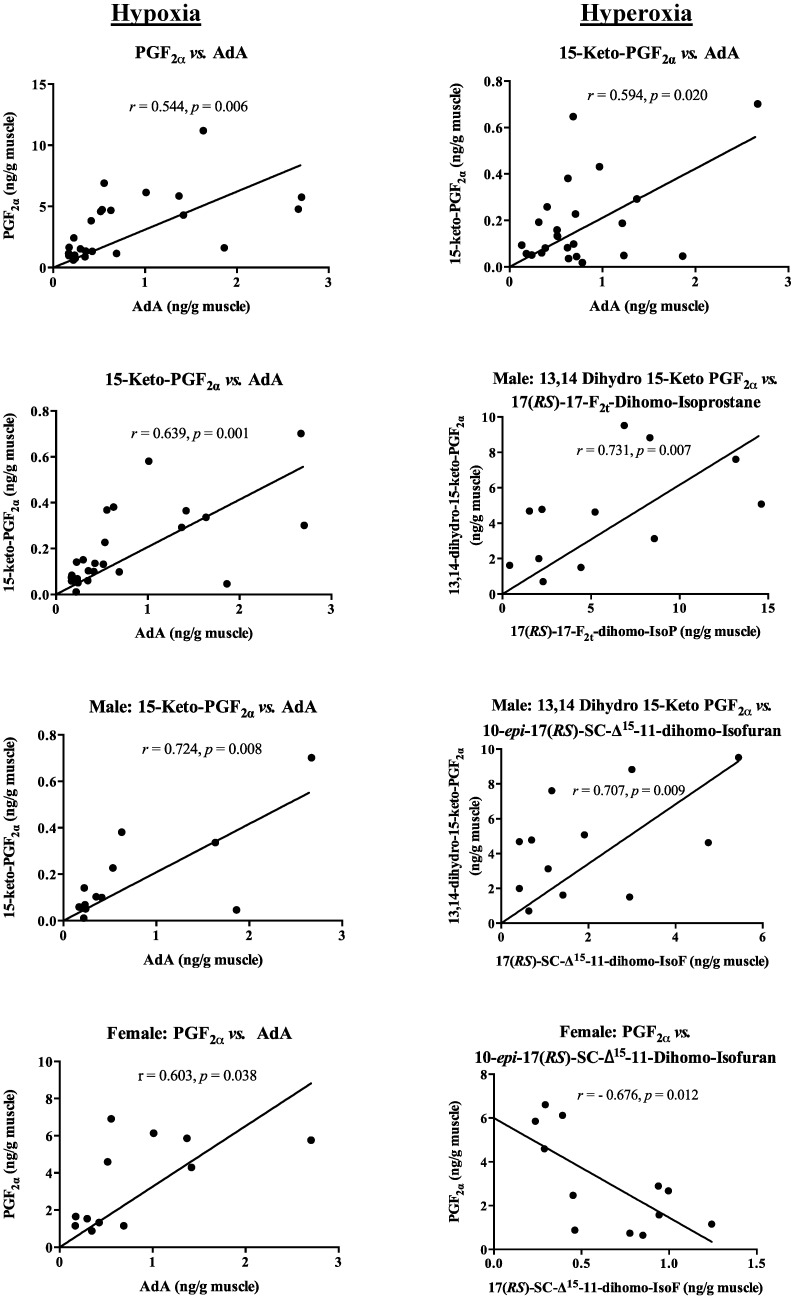
Pearson’s correlation of adrenic acid (AdA) and enzyme-independent oxygenated metabolites, and pheromone mediators after hypoxia and hyperoxia exposure. Each point represents one fish.

It appears that the role of AdA is vital in the generation of pheromone mediators under oxidative stress. Under hypoxia condition, strong positive correlation was identified between AdA and the pheromone mediators in male and female fishes (Figure 4). However in hyperoxia condition, the enzyme-independent oxidized products of AdA namely 17(*RS*)-17-F_2t_-dihomo-isoprostane and 10-*epi*-17(*RS*)-SC-Δ^15^-11-dihomo-isofuran had a strong association with pheromone mediators in male fishes whereas a negative association was recorded for female fishes (Figure 4). No significant correlation was found between AA, EPA or DHA, and the pheromone mediators, and the generation of enzyme-dependent oxidized lipid products and the pheromone mediators.

**Table 1:** Concentrations of isoprostanoids from non-enzymatic peroxidation of polyunsaturated fatty acids in fish muscles after hypoxia and hyperoxia exposure.

		Normoxia	Hypoxia			Hyperoxia		
Time (h)		0	1	6	*p-trend*	1	6	*p-trend*
*Arachidonic Acid*								
15-F_2t_-IsoP	All	2.20 ± 0.76	2.44 ± 1.49	2.93 ± 0.90	*0.727*	1.26 ± 0.26	5.68 ± 2.24	*0.078*
	M	1.51 ± 0.58	3.46 ± 2.90	2.33 ± 0.65	*0.738*	1.24 ± 0.39	7.24 ± 4.23	*0.210*
	F	2.88 ± 1.43	3.41 ± 1.40	3.54 ± 1.77	*0.951*	1.29 ± 0.40	4.12 ± 1.94	*0.398*
2,3-dinor-15-F_2t_-IsoP^+^	All	1.89 ± 1.03	0.32 ± 0.06	0.76 ± 0.27	*0.202*	0.94 ± 0.25	0.63 ± 0.20	*0.349*
	M	2.68 ± 2.13	0.27 ± 0.02	0.80 ± 0.32	*0.394*	1.24 ± 0.38	0.57 ± 0.20	*0.505*
	F	1.10 ± 0.14	0.36 ± 0.12	0.72 ± 0.47	*0.258*	0.64 ± 0.29	0.72 ± 0.47	*0.504*
2,3-dinor-5,6-dihydro-15-F_2t_-IsoP^+^	All	19.43 ± 8.34	7.54 ± 1.39	7.96 ± 0.75	*0.177*	23.00 ± 8.27	13.84 ± 4.31	*0.670*
	M	25.01 ± 16.30	0.27 ± 0.02	7.98 ± 1.44	*0.384*	29.00 ± 14.67	16.85 ± 8.58	*0.816*
	F	13.16 ± 6.34	7.13 ± 2.03	7.95 ± 0.73	*0.440*	17.00 ± 8.94	10.83 ± 2.61	*0.803*
*Adrenic Acid*								
7(*RS*)-7-F_2t_-dihomo-IsoP	All	0.98 ± 0.30	0.47 ± 0.09	0.50 ± 0.09	*0.118*	0.63 ± 0.15	0.56 ± 0.13	*0.321*
	M	0.81 ± 0.43	0.49 ± 0.19	0.36 ± 0.09	*0.537*	0.68 ± 0.22	0.79 ± 0.17	*0.952*
	F	1.17 ± 0.46	0.45 ± 0.06	0.64 ± 0.13	*0.218*	0.59 ± 0.25	0.33 ± 0.11	*0.198*
17(*RS*)-17-F_2t_-dihomo-IsoP	All	3.44 ± 1.43	2.37 ± 0.37	3.05 ± 0.54	*0.706*	3.99 ± 0.87	5.78 ± 1.45	*0.409*
	M	4.33 ± 2.97	2.43 ± 0.61	2.13 ± 0.34	*0.646*	4.95 ± 1.64	8.15 ± 2.31	*0.501*
	F	2.55 ± 0.42	2.31 ± 0.52	3.98 ± 0.83	*0.173*	3.02 ± 0.44	3.44 ± 0.86	*0.604*
*Eicosapentaenoic Acid*								
8-F_3t_-IsoP	All	2.95 ± 0.53	2.58 ± 0.57	2.37 ± 0.33	*0.705*	2.75 ± 0.64	3.73 ± 0.92	*0.598*
	M	3.03 ± 0.90	2.51 ± 1.05	2.65 ± 0.63	*0.912*	3.77 ± 0.98	4.48 ± 1.58	*0.701*
	F	2.86 ± 0.70	2.65 ± 0.63	2.10 ± 0.23	*0.626*	1.74 ± 0.52	2.99 ± 1.04	*0.492*
*Docosahexaenoic Acid*								
4(*RS*)-4-F_4t_-NeuroP	All	61.07 ± 11.80	71.44 ± 28.17	33.77 ± 6.79	*0.284*	61.07 ± 11.80	77.33 ± 26.96	*0.766*
	M	54.21 ± 7.74	73.51 ± 49.02	37.96 ± 12.63	*0.706*	59.27 ± 19.80	97.96 ± 50.16	*0.580*
	F	70.95 ± 6.41	69.37 ± 36.00	29.58 ± 6.63	*0.347*	62.87 ± 15.98	56.72 ± 24.35	*0.845*
10-F_4t_-NeuroP	All	1.54 ± 0.21	1.64 ± 0.50	0.94 ± 0.32	*0.356*	1.59 ± 0.25	1.01 ± 0.22	*0.164*
	M	1.06 ± 0.13	0.94 ± 0.32	1.21 ± 0.59	*0.894*	1.35 ± 0.35	1.04 ± 0.44	*0.769*
	F	2.01 ± 0.22 **	2.34 ± 0.85	0.68 ± 0.27	*0.119*	1.85 ± 0.37	0.99 ± 0.16	*0.049*

Values are mean ± SEM ng/g muscle except + μg/g muscle, *n* = 8 for all and *n* = 4 for each gender group. M: male; F: female; IsoP: isoprostane; NeuroP: neuroprostane; One-way ANOVA for linear *p*-trend is annotated for the effect of oxygen tension change with time. ** *p* < 0.01 M *vs*. F.

**Table 2:** Concentration of lipoxygenase-mediated oxidized lipid products of polyunsaturated fatty acids in fish muscles after hypoxia and hyperoxia exposure.

		Normoxia	Hypoxia			Hyperoxia		
Time (h)		0	1	6	*p-trend*	1	6	*p-trend*
*Arachidonic Acid*								
5(*S*)-HETE	All	41.90 ± 4.98	31.05 ± 11.44	39.10 ± 7.06	*0.672*	26.20 ± 4.98	49.40 ± 12.38	*0.188*
	M	29.62 ± 2.17	16.19 ± 3.63	40.53 ± 8.07	*0.029*	30.10 ± 9.16	52.25 ± 16.99	*0.312*
	F	54.17 ± 12.12	45.91 ± 21.21	37.66 ± 12.87	*0.771*	22.29 ± 4.66	46.54 ± 20.53	*0.294*
8(*S*)-HETE	All	25.70 ± 5.66	26.08 ± 7.66	11.19 ± 1.93	*0.126*	30.79 ± 4.43	21.88 ± 3.96	*0.425*
	M	26.41 ± 7.29	17.72 ± 7.72	10.84 ± 2.99	*0.274*	33.95 ± 7.62	25.92 ± 7.59	*0.707*
	F	25.00 ± 9.78	34.43 ± 12.94	11.54 ± 2.90	*0.282*	27.64 ± 5.18	17.85 ± 2.14	*0.566*
12(*S*)-HETE	All	161.96 ± 62.88	157.03 ± 50.60	69.20 ± 19.23	*0.325*	203.29 ± 48.19	180.55 ± 53.86	*0.870*
	M	173.00 ± 90.36	127.50 ± 47.59	73.66 ± 26.64	*0.538*	246.30 ± 91.76	259.30 ± 94.76	*0.756*
	F	150.9 ± 101.00	186.50 ± 95.41	64.73 ± 31.65	*0.579*	160.30 ± 34.63	101.80 ± 20.51	*0.783*
15(S)-HETE	All	2.53 ± 0.29	13.00 ± 6.10	5.44 ± 2.55	*0.160*	1.79 ± 0.32	12.23 ± 4.85 *	*0.027*
	M	2.66 ± 0.40	127.50 ± 47.59	7.63 ± 5.09	*0.543*	1.75 ± 0.45	15.33 ± 8.74	*0.781*
	F	2.40 ± 0.48	186.50 ± 95.41	3.25 ± 1.08	*0.332*	1.82 ± 0.52	9.13 ± 5.18	*0.209*
*Eicosapentaenoic Acid*								
RvE1	All	0.32 ± 0.04	0.21 ± 0.07	0.33 ± 0.12	*0.533*	0.17 ± 0.03	0.23 ± 0.04	*0.027*
	M	0.26 ± 0.04	0.12 ± 0.01	0.28 ± 0.16	*0.446*	0.13 ± 0.05	0.27 ± 0.05	*0.142*
	F	0.39 ± 0.07	0.30 ± 0.12	0.38 ± 0.20	*0.894*	0.20 ± 0.04	0.19 ± 0.05	*0.045*
*Docosahexaenoic Acid*								
RvD1	All	1.15 ± 0.15	0.71 ± 0.11	2.91 ± 1.69	*0.265*	0.83 ± 0.09	0.72 ± 0.15	*0.073*
	M	1.02 ± 0.22	0.66 ± 0.12	1.88 ± 0.90	*0.304*	0.82 ± 0.11	0.69 ± 0.26	*0.550*
	F	1.28 ± 0.20	0.76 ± 0.20	3.95 ± 3.43	*0.505*	0.85 ± 0.15	0.75 ± 0.19	*0.132*

Values are mean ± SEM ng/g muscle *n* = 8 for all and *n* = 4 for each gender group. M: male, F: female. One-way ANOVA for linear *p*-trend is annotated for the effect of oxygen tension change with time. HETE: hydroxyeicosatetraenoic acid; RvEI: resolvin E1; RvD1: resolvin D1. * *p* < 0.05 1 h *vs*. 6 h and normoxia *vs*. 6 h.

### 2.5. Effect of Hypoxia-Hyperoxia on Reproductive Behavior and Discharge of Pheromone Mediators in Water

**Figure 5:**
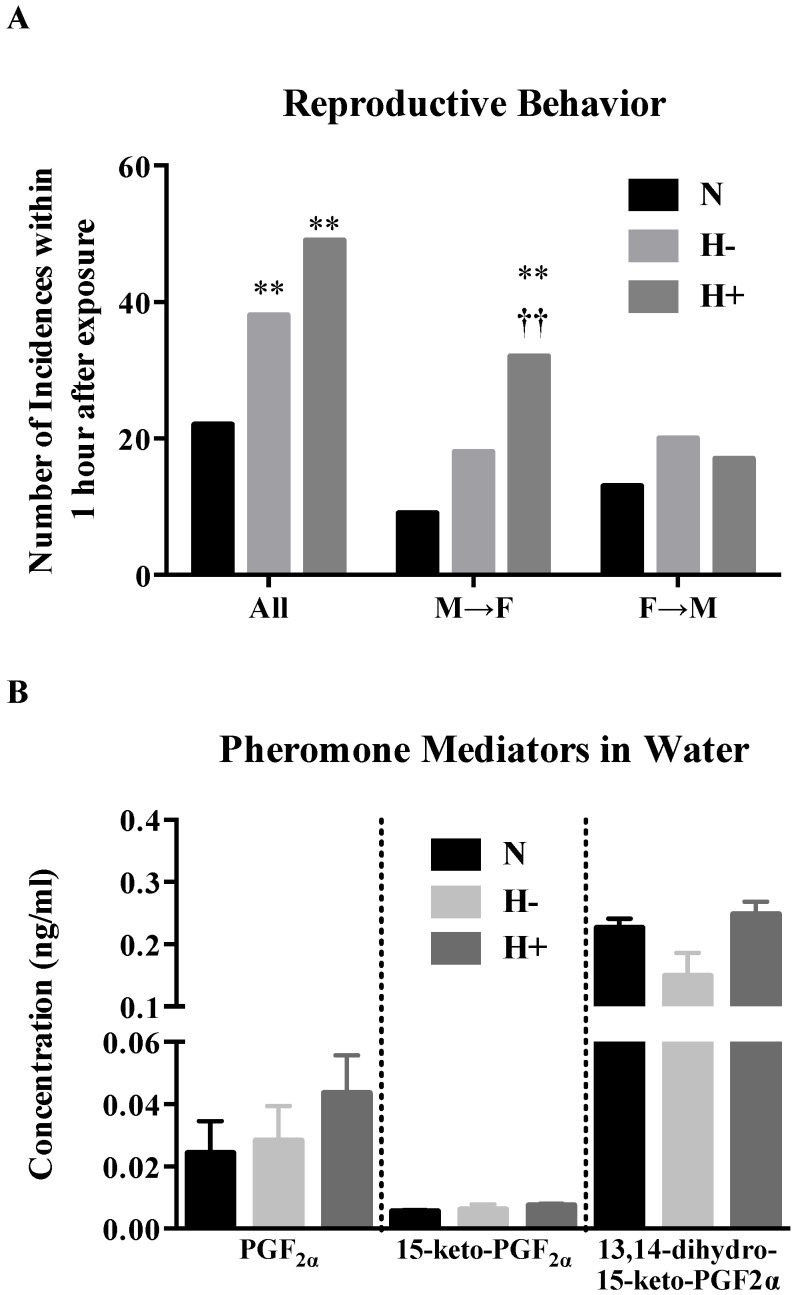
Reproductive behaviors (**A**) and pheromone mediator concentrations (**B**) in marine water after exposure to oxidative stress. Male (M) and female (F) fishes were contained in the same tank and exposed to hypoxia or hyperoxia for 6 h. Values (*n* = 4 pairs) indicate number of incidences within one hour after exposure. F→M: female approach male; M→F: male approach female; N: normoxia; H−: hypoxia; H+: hyperoxia. *χ*^2^-test indicates significant ** *p* < 0.01 *vs*. normoxia and ^††^ *p* <0.01 M→F *vs*. normoxia and hypoxia.

In order to test the reproductive behaviors under oxidative stress two actions, the pecking gestures and following movements of the marine fishes were investigated. Both hypoxia and hyperoxia conditions increased reproductive behavior (Figure 5A) compared to normoxia. However, in hyperoxia, the male fishes contributed to 85% of the movements. Visual observations also showed sluggish movement of the fishes after hypoxia treatment, whereas aggressive movements especially of male fish were shown after hyperoxia treatment compared to the normoxic state. This behavior did not appear to be related to the pheromone mediators released in the water. As shown in Figure 5B, change in oxygen tension did not alter the concentration of free pheromone mediators released in the water after six hours of treatment.

## 3. Discussion 

In this study we evaluated the effect of hypoxia and hyperoxia environments on PUFA metabolism in marine fish. This is a follow-up study of our previous findings in which the presence of exogenous hydrogen peroxide in marine water reduced PGF_2α_ and PUFA levels, and induced production of oxidized lipid products [6]. In this investigation we addressed the impact of oxidative stress on important omega-3 and omega-6 PUFAs in marine fish by measuring novel oxidized lipid products. These products included isoprostanoids, the PGF_2α_ pheromone mediators and its derivatives, and more recently identified isofuranoids, which have come to attention for their dominance in the hyperoxia environment *in vivo* [12,13]. 

Evaluation of oxidized lipid products particularly isoprostanoids as biomarkers for *in vivo* assessment is well defined and most of them are generated by non-enzymatic related ROS reaction with PUFA [13,14]. This biomarker of oxidative stress has been well studied in humans where AA is the common omega-6 PUFA in the body and DHA is probably the most omega-3 PUFA concentrated in the brain. However in fish body, this might be different, since one of the most common PUFAs is DHA and with low concentration of AA and probably AdA. Nevertheless in humans, it is common to assess products of AA *i.e.*, 15-F_2t_-isoprostane [7,14] whereas for more specialized conditions such as neurological disorders, 4(*RS*)-4-F_4t_-neuroprostane from DHA and 17(*RS*)-17-F_2t_-dihomo-isoprostane from AdA is assessed [12,13,15,16]. Furthermore, it is known under high oxygen tension, release of isofurans from AA [17,18], 10-*epi*-17(*RS*)-SC-Δ^15^-11-dihomo-isofuran from AdA [19] and neurofurans from DHA [20] predominate. Nonetheless to date, oxidized products of AdA have been insufficiently investigated in different biological systems.

The dynamics of hypoxia and hyperoxia environment also depends on the cleanliness of the marine water and the climate; environmental pollutants such as contaminants from fertilizers and industrial waste, and global warming concomitantly disrupt the oxygen balance in marine water [1,4,21]. Our study showed exogenous change of oxygen concentration in marine water was not as detrimental as extrinsic chemical insult. It was surprising to find hypoxia or hyperoxia conditions did not influence the generation of oxidized lipid products from PUFA mainly 15-F_2t_-isoprostane, 8-F_3t_-isoprostane, 4(*RS*)-4-F_4t_-neuroprostane, and a majority of LOX-mediated products in the fish. The findings were opposed to our previous report where exposure to a chemical oxidant such as hydrogen peroxide induced oxidized lipid products and depleted its precursor PUFA [6]. Furthermore, only recently F_2_-isoprostanes and neuroprostanes levels were assessed in the brain of zebrafish exposed to acute hypoxia but no significant change was reported [22]. 

**Figure 6:**
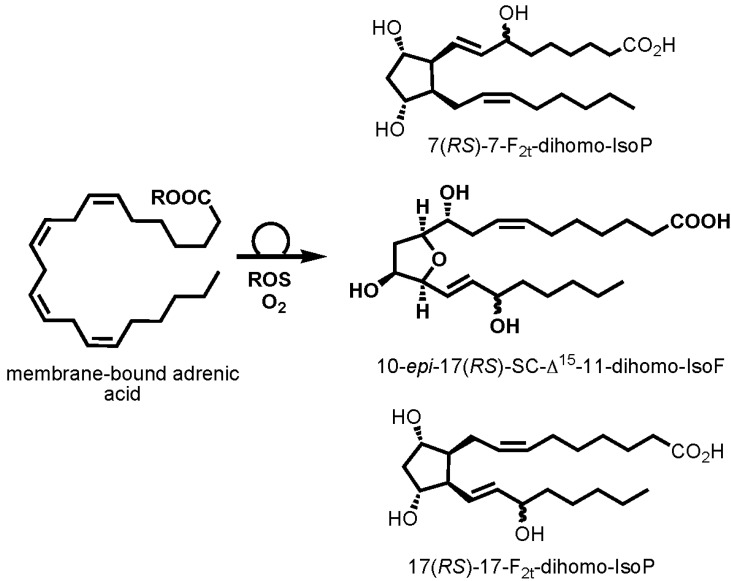
Structure of adrenic acid and its enzyme-independent oxidation products, 7(*RS*)-7-F_2t_-dihomo-IsoP, 17(*RS*)-17-F_2t_-dihomo-IsoP and 10-*epi*-17(*RS*)-SC-Δ^15^-11-dihomo-IsoF identified in fish muscle after oxidative stress exposure. IsoP: isoprostane; IsoF: isofuran.

It should be noted that the generation of PGF_2α_ and the derivatives in fish are associated with pheromones produced in the ovaries or reproductive organs and released into the water. However, the role of the prostaglandins is not limited to these, and in part act as a mediator for hormonal regulation in reproduction and smooth muscle contraction. In our study, it was anticipated that the release of prostaglandin pheromone mediators would be modified by the hypoxia and hyperoxia environments since it is known that the hypoxic state reduces the rate of fertility and hatching in medaka [1]. Instead such observation was not made, and rather the generation of AdA and its oxygenated metabolites showed an impact on prostaglandin pheromone mediators. It is clear hyperoxia in marine fish affected AdA metabolism. Specifically, the association of its oxidized lipid products 17(*RS*)-17-F_2t_-dihomo-isoprostane and 10-*epi*-17(*RS*)-SC-Δ^15^-11-dihomo-isofuran, and the pheromone mediators in male fish stimulated active reproductive behaviors. This also concurs with the use of hyperoxic environment in fish hatchery for enhancing survival and growth of larvae in the early stages of development [23]. However in this study, only up to six hours treatment of hypoxia or hyperoxia was given to the fish, therefore the outcome is not known after prolonged treatment or after a constant change of water as how it would be in the actual marine ecosystem. Without doubt 17(*RS*)-17-F_2t_-dihomo-isoprostane, 10-*epi*-17(*RS*)-SC-Δ^15^-11-dihomo-isofuran and AdA appear to take part in fish (patho)physiology in this study as well as in pig brain, and rat heart and brain as shown in our previous studies [19,24]. Despite the relatively low concentration of AdA compared to AA, DHA and EPA, it seems to be sensitive to enzyme-independent peroxidation and the metabolites generated (Figure 6) greatly influenced the fish endocrine system.

It would be interesting to investigate if 17(*RS*)-17-F_2t_-dihomo-isoprostane or 10-*epi*-17(*RS*)-SC-Δ^15^-11-dihomo-isofuran indeed contribute to the reproductive behavior by microinjecting these compounds to the reproductive system of the male fish to test the pheromone effect towards female fish. Moreover, it should also be noted that the role of Δ^5^-desaturase enzyme in PUFA metabolism [25] might be inhibited in the omega-3 PUFA pathway as there is a tendency for decreased EPA under oxidative stress or the rate of β-oxidation under hyperoxia is enhanced and used for energy during the swimming activity. As a follow-up, it would be appropriate to understand the benefits of this phenomenon in the epigenetic approach; induce repeated hyperoxia-normoxia-hyperoxia conditions to manipulate pheromone production to observe the fish population growth and sustainability.

## 4. Materials and Methods

### 4.1. Treatment of Medaka Fish

Adult marine medaka fishes (*Oryzias melastigma*) 10 months old were maintained in fully aerated water set at photoperiod of 14 h light/10 h dark, water temperature at 26 ± 1 °C, ammonia level (0.010 ± 0.005 mg/L) and pH 8. The fishes were fed twice daily with frozen artemia and hormone-free flake food (AX5, Aquatic Eco-Systems Inc., Apopka, FL, USA) prior to exposure to hypoxia or hyperoxia, and the water was cleaned (50% changed) every 72 h.

Medaka fish (*n* = 8) were paired (1:1, male to female) per treatment and placed in separate water tanks of 3 L marine water. A hypoxia condition (dissolved oxygen 2.0–3.0 mg/L) was established by pumping nitrogen gas for 5 min and a hyperoxia condition (dissolved oxygen 8.0–9.0 mg/L) was established by pumping air into water. The water oxygen level was assessed using the dissolved oxygen monitor (Pinpoint II, American Marine Inc., Ridgefield, CT, USA). The medaka fishes were exposed to hypoxia or hyperoxia conditions for 1 h and 6 h following our previous report [6]. A normoxia condition was established by placing medaka fish in fish tanks without an air pump (dissolved oxygen 6.0–7.0 mg/L) and served as control. After treatment in different oxygen levels, each medaka fish was anesthetized on ice and then sacrificed. All the internal organs were removed. The body muscle was washed by phosphate buffered saline (PBS, pH 7.4) with indomethacin and butylated hydroxytoluene (BHT), and stored at −80 °C for further lipid analysis. Volumes (50 mL) of water samples were taken at 6 h time points and stored at −80 °C for free prostaglandin pheromones analysis.

### 4.2. Sample Preparation for Lipid Extraction

In this study we measured the lipids of the body muscle and not the liver (the deposit for fats and lipids), as we were also investigating the body pheromone mediators level after the treatments. The main body muscle (0.6–0.8 g) excluding the head and internal organs was homogenized in Folch solution with BHT (0.005%). Sodium chloride solution (2 mL, 0.9% w/v) was added to the homogenized samples thereafter centrifuged at 800× *g* for 10 min at 4 °C. The lower organic layer was transferred to a glass vial and the solvent was evaporated under a stream of nitrogen gas. The dried Folch extract was hydrolyzed with PBS (pH 7.4) and potassium hydroxide (1 M) prepared in methanol (1:1) at room temperature overnight in the dark. After hydrolysis 40 mM formic acid buffer, methanol, and hydrochloric acid were added before solid phase extraction. For solid phase extraction (SPE), 60 mg MAX columns (Oasis, Waters, Milford, MA, USA) was used to purify the hydrolyzed samples according to the Chung *et al.* [6] method. In brief, the column was conditioned with 20 mM formic acid, washed with 2% ammonium hydroxide, hexane and hexane with ethyl acetate (70:30) and thereafter eluted with hexane, ethanol, and acetic acid mix (70.00:29.98:0.02). The solvent from the extracted samples was evaporated under a stream of nitrogen gas and the dried extract was re-dissolved in methanol containing internal standards obtained from Cayman Chemicals (Ann Arbor, MI, USA) and IBMM (Montpellier, France) for fatty acids, oxidized lipid products, and pheromones analysis. The same SPE regime was used for purifying 20 mL of marine water collected at the 6 h time point for measurement of free prostaglandin pheromones.

### 4.3. Analysis of Oxidized Lipid Products of Fatty Acids

Oxidized lipid products, 15-F_2t_-isoprostane, 2,3-dinor-15-F_2t_-isoprostane, 2,3-dinor-5,6-dihydro-15-F_2t_-isoprostane, 8-F_3t_-isoprostane, 4(*RS*)-4-F_4t_-neuroprostane, 10-F_4t_-neuroprostane, 7(*RS*)-7-F_2t_-dihomo-isoprostane, 17(*RS*)-17-F_2t_-dihomo-isoprostane, isofurans, 10-*epi*-17(*RS*)-SC-Δ^15^-11-dihomo-isofuran, neurofurans, LOX-mediated HETEs and resolvins (RvD1 and RvE1), and PUFAs (AA, AdA, EPA and DHA) analyses were performed using a 1290 Infinity LC system (Agilent, Santa Clara, CA, USA) according to [6,19] with modification. A Hillic C18 column (2.4 mm particle size, 50 × 2.4 mm, Phenomenex, Torrance, CA, USA) was used and maintained at 30 °C. The mobile phase consisted of 0.1% acetic acid in water (A) and 0.1% acetic acid in methanol (B) and was set to 100 μL/min flow rate. The mobile phase ran in a linear gradient from phase A 50% to phase B 50% over 3 min, B 95% to A 5% for 0.5 min and B 55% to A 45% for 3 min. The sample injection volume was 20 μL. A QTrap 3200 triple quadrupole mass spectrometer (MS/MS, Sciex Applied Biosystems, Carlsbad, CA, USA) was operated in the negative atmospheric pressure chemical ionization (APCI) mode with a source temperature of 500 °C and capillary temperature of 250 °C. The declustering and entrance potential, and the collision energy were set according to our previous reports to maximize the ion currents of the precursor to product ion dissociation. The spray voltage was set to −4000 V and nitrogen gas was used as curtain gas. 

The analytes were detected by MS/MS using multiple reactions monitoring (MRM). Quantitation of each analyte was achieved by relating the peak area with its corresponding internal standard peak. Heavy labeled isotope AA-d_8_, EPA-d_5_, DHA-d_5_, 15-F_2t_-isoprostane-d_4_, 4(*RS*)-4-F_4t_-neuroprostane-d_4_, 10-F_4t_-neuroprostane-d_4_, 5(*S*)-HETE-d_8_, 12(*S*)-HETE-d_8_, and 15(*S*)-HETE-d_8_ were used for quantitation of the respective compounds. Concentration of 7(*RS*)-7-F_2t_-dihomo-isoprostane, 17(*RS*)-17-F_2t_-dihomo-isoprostane, 8-F_3t_-isoprostane, isofurans, 10-*epi*-17(*RS*)-SC-Δ^15^-11-dihomo-isofuran and RvE1 were determined using 15-F_2t_-isoprostane-d_4_, neurofurans and RvD1 using 4(*RS*)-4-F_4t_-neuroprostane-d_4_, and 8(*S*)-HETE using 5(*S*)-HETE-d_8_ as internal standard and where possible, the ratio of response to the heavy labeled isotope was pre-determined with the non-deuterated pure compounds for final concentration calculation. 

### 4.4. Pheromone Mediator Analysis

The muscle lipid and water extracts from solid phase extraction were tested for the pheromones, PGF_2α_, 15-keto-PGF_2α_ and 13,14-dihydro-15-keto-PGF_2α_ concentrations using LC-MS/MS. Similar settings of the LC-MS/MS were made as for oxidized lipid products except for the declustering and entrance potentials, collision energy, and precursor and product ions of dissociation. Heavy labeled isotope PGF_2α_-d_4_ and 13,14-dihydro-15-keto-PGF_2α_-d_4_ were used for quantitation of the respective compounds, and PGF_2α_-d_4_ for 15-keto-PGF_2α_.

### 4.5. Reproductive Behavior

After treatment of hypoxia and hyperoxia exposure for 6 h, the behaviors of the marine fishes in male and female pairs (*n* = 4 pairs) per treatment were video recorded (Sony, Tokyo, Japan) for 1 h and the total number of behavioral occurrences was recorded. From the list of behaviors [12], two common types were identified which included, following and pecking. For following behavior, a pair of fish orientates toward each other and stays in parallel. Both swim slowly, approach, and tail each other. For pecking behavior the pair of fish peck-up each other and toss up their belly by staying at the bottom with their mouths to orientate under one another. 

### 4.6. Statistical Analysis

Statistical analysis was performed by GraphPad Prism version 6.0 for Macintosh (GraphPad Software, La Jolla, CA, USA). All values are expressed as mean ± SEM. Any significant changes on analysis of variance (mixed model) for the treatment were further analyzed by unpaired Student’s *t*-test. Pearson’s correlation was tested between the pheromones and the analytes in male and female marine fish separately. Chi-square (*χ*^2^) test was adapted for the reproductive behavioral test.

## 5. Conclusions

This study displayed the generation of a comprehensive set of oxidized lipid products of omega-3 and omega-6 polyunsaturated fatty acids (PUFA) rich marine fish under natural environmental oxidative stress exposure and its effect on pheromone mediators. It is conspicuous that AdA and its metabolites 17(*RS*)-17-F_2t_-dihomo-isoprostane and 10-*epi*-17(*RS*)-SC-Δ^15^-11-dihomo-isofuran had an impact on male fish pheromone mediation and reproductive behavior when under oxidative stress. Our findings also further indicate the importance of AdA and the oxidized lipid products of PUFA in biological systems in evaluating oxidative stress whether as a biomarker or as bioactive compounds.

## Abbreviation

2,3-dinor-15-F_2t_-IsoP: 2,3-dinor-15-F_2t_-isoprostane
2,3-dinor-5,6-dihydro-15-F_2t_-IsoP: 2,3-dinor-5,6-dihydro-15-F_2t_-isoprostane
4(*RS*)-4-F_4t_-NeuroP: 4(*RS*)-4-F_4t_-neuroprostane
7(*RS*)-7-F_2t_-dihomo-IsoP: 7(*RS*)-7-F_2t_-dihomo-isoprostane
8-F_3t_-IsoP: 8-F_3t_-isoprostane
10-F_4t_-NeuroP: 10-F_4t_-neuroprostane
13,14-dihydro-15-keto-PGF_2α_: 13,14-dihydro-15-keto prostaglandin F_2α_
15-F_2t_-IsoP: 15-F_2t_-isoprostane
15-keto-PGF_2α_: 15-keto-prostaglandin F_2α_
17(*RS*)-17-F_2t_-dihomo-IsoP: 17(*RS*)-17-F_2t_-dihomo-isoprostane
10-*epi*-17(*RS*)-SC-Δ^15^-11-dihomo-IsoF: 10-*epi*-17(*RS*)-SC-Δ^15^-11-dihomo-isofuran
AA: arachidonic acid
AdA: adrenic acid
DHA: docosahexaenoic acid
EPA: eicosapentaenoic acid
HETE: hydroxyeicosatetraenoic acid products
IsoFs: isofurans
LC-MS/MS: liquid chromatography tandem mass spectrometry
LOX: lipoxygenase
NeuroFs: neurofurans
PGF_2α_: prostaglandin F_2α_
RvD1: resolvin D1
RvE1: resolvin E1
